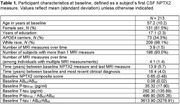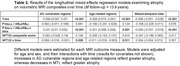# The synaptic marker NPTX2 and longitudinal brain atrophy among cognitively unimpaired adults

**DOI:** 10.1002/alz.093019

**Published:** 2025-01-09

**Authors:** Claire L Anderson, Juan Vazquez, Yuxin Zhu, Corinne Pettigrew, Abhay Moghekar, Guray Erus, Christos Davatzikos, Mei‐Fang Xiao, Paul F Worley, Marilyn S. Albert, Anja Soldan

**Affiliations:** ^1^ Johns Hopkins University School of Medicine, Baltimore, MD USA; ^2^ Johns Hopkins Bloomberg School of Public Health, Baltimore, MD USA; ^3^ University of Pennsylvania, Philadelphia, PA USA; ^4^ Artificial Intelligence in Biomedical Imaging Laboratory (AIBIL), Center for and Data Science for Integrated Diagnostics (AI2D), Perelman School of Medicine, University of Pennsylvania, Philadelphia, PA USA

## Abstract

**Background:**

Previous studies have shown that higher levels of the synaptic protein NPTX2, measured in cerebrospinal fluid (CSF), are associated with lower risk of progression from normal cognition to Mild Cognitive Impairment (MCI) and dementia. Among those with MCI or dementia, higher NPTX2 levels have been associated with decreased atrophy in AD‐vulnerable brain regions. It is not known, however, whether NPTX2 levels influence brain atrophy among cognitively unimpaired participants. This study examined the association between baseline CSF NPTX2 and longitudinal MRI brain volumetric measures among participants who were cognitively unimpaired at baseline, and whether AD pathology levels modify this relationship.

**Methods:**

CSF and MRI data were collected from 213 BIOCARD Study participants (M baseline age = 57.2y, mean MRI follow‐up=13.9y), including 41 who have since progressed to MCI/dementia (Table 1). The analysis focused on a z‐transformed composite measure of three NPTX2 peptides, measured using parallel reaction monitoring mass spectroscopy. CSF Ab_1‐42_/Ab_1‐40_ and p‐tau_181_ were measured using the Fujirebio Lumipulse G1200 assays, with levels of AD pathology summarized as the p‐tau_181_/(Ab_1‐42_/Ab_1‐40_) ratio_._ Atrophy on volumetric MRI scans was quantified with MUSE and assessed as three composites including: (1) AD vulnerable regions (SPARE‐AD), (2) medial temporal lobe (MTL) regions, and (3) regions indexing advanced non‐AD related brain aging (SPARE‐BA).

**Results:**

In mixed‐effects models covarying age, sex, and AD pathology levels (i.e., p‐tau_181_/(Ab_1‐42_/Ab_1‐40_)), higher baseline NPTX2 levels were associated with less atrophy over time in AD vulnerable (β=‐.008, p=0.03) and in age‐related regions (β=‐.01 p=0.01), see Table 2. Higher AD‐biomarker levels were associated with greater longitudinal atrophy in all MRI composites (all p<=0.001) but did not moderate the association between NPTX2 and atrophy (all p>0.52 for interactions of NPTX2 x p‐tau_181_/(Ab_1‐42_/Ab_1‐40_) x time).

**Conclusions:**

These findings suggest that among cognitively unimpaired individuals, higher levels of NPTX2 may slow longitudinal atrophy in AD‐vulnerable and age‐related regions, independently of AD pathology levels. As a non‐AD‐specific marker of synaptic and neuronal function, these findings support the view that NPTX2 may be a resilience factor against neurodegeneration and cognitive decline and suggest that developing novel therapeutics that target NPTX2 and related synaptic proteins may have broad applicability.